# Women’s lived experience of intimate partner violence manifestations during the breastfeeding period: a lifeworld hermeneutic study

**DOI:** 10.1186/s13006-024-00690-5

**Published:** 2024-12-16

**Authors:** Ida Gustafsson, Katarina Karlsson, Aleksandra Jarling, Lina Palmér

**Affiliations:** 1 Faculty of Caring Science, Work Life and Social Welfare, University of Borås, Borås, 501 90 Sweden; 2 PreHospen Centre for Prehospital Research, University of Borås, Borås, 501 90 Sweden

**Keywords:** Breastfeeding, Caring, Continuum of violence, Lived experience, Intimate Partner Violence (IPV), Lifeworld hermeneutics, Qualitative research, Women

## Abstract

**Background:**

One in three women will experience Intimate Partner Violence (IPV). Exposure during breastfeeding endangers women’s and children’s health and wellbeing, negatively affects breastfeeding, and violates human rights and global sustainability goals. Previous qualitative studies have demonstrated that existential aspects are crucial in the separate experience of both IPV and breastfeeding. However, there is a lack of studies examining the meaning of the concurrent experience of these phenomena. An enhanced understanding of the experience of IPV manifestations during the breastfeeding period may inform the provision of care and support for women exposed to IPV. Accordingly, the study aims to explain and understand women’s lived experience of IPV manifestations during the breastfeeding period.

**Methods:**

The study adopts a lifeworld hermeneutic approach based on Reflective Lifeworld Research. Data collection was conducted between June 2022 and August 2023. Swedish women with experience of the phenomenon *IPV manifestations during the breastfeeding period* participated either through written lifeworld stories (forty-nine women) or lifeworld interviews (nine women). Data were analysed interpretatively. The main interpretation was inspired by Liz Kelly’s theory ‘*The continuum of violence’*.

**Results:**

The results show that women experience IPV manifestations during breastfeeding in terms of being *accused*,* devalued*,* neglected*,* controlled*,* opposed*,* forced to adapt*, and/or *punished*. The main interpretation suggests that the manifestations are intertwined within a multidimensional continuum where the most frequent IPV manifestations are less commonly recognised as violence. The main interpretation further illustrates that the continuum is dependent on both the subjective lifeworld of the woman and the patriarchal context in which it exists. In relation to the patriarchal context, the breastfeeding intimacy within the mother–child dyad is pivotal to explaining and understanding the phenomenon.

**Conclusions:**

The breastfeeding intimacy within the mother–child dyad seems to change the intersubjective power balance in the partner relationship and provoke partners, making breastfeeding women especially vulnerable to IPV. Knowledge of breastfeeding women’s lived experience of exposure to IPV is central for carers to strengthen their ability to support women’s health and wellbeing.

## Background

Globally, almost one in three women experience physical or sexual Intimate Partner Violence (IPV) during their lifetime [1]. Nordic countries are at the forefront of gender equality, but Sweden still reports relatively high IPV frequency [2]. IPV is defined by the World Health Organisation (WHO) as *behaviour by an intimate partner or ex-partner that causes physical*,* sexual*,* or psychological harm*,* including physical aggression*,* sexual coercion*,* psychological abuse*,* and controlling behaviours* [3]. IPV could be physical, emotional, economical, material, social, and/or digital [4]. Women are more often exposed to IPV than men, especially sexual and repeated violence [5]. According to WHO, violence against women is a multifaceted and gender-based global threat to human rights and public health, existing both at individual and societal levels. IPV poses a threat to women’s and children’s health and wellbeing, human rights, and global sustainability goals [3]. Societal IPV health costs are huge [6, 7] and negative health effects persist for many years [8]. Women risk acute and chronic physical, mental, gynaecological, and reproductive health issues [9]. They are twice as likely as other women to suffer from depression or addiction and are at risk of premature death [3]. There are statistical associations between depression and a negative impact on breastfeeding [10], and reduced rates of depression in breastfeeding women have also been demonstrated [11], but further research is needed.

Reported IPV incidence during childbearing varies globally [12–15], as do the validity of existing studies. It is unclear whether IPV incidence increases during pregnancy and the postpartum period [12, 15], or whether a statistical correlation between IPV and breastfeeding exists [16]. However, women exposed to IPV tend to be less likely to initiate breastfeeding [17] and tend to breastfeed for shorter durations [17, 18]. Lower breastfeeding rates risks negative health effects for women and children. At the population level, non-breastfed children are at increased risk of infection, obesity, and diabetes, and non-breastfeeding women are more likely to suffer from breast- and ovarian cancers [19]. Increased breastfeeding rates correspond globally to Agenda 2030’s sustainability goals of *reduced hunger*,* improved health and wellbeing*,* sustainable consumption*, and *reduced climate change* [20].

Qualitative research has demonstrated that breastfeeding is not only a source of nutrition, but an existential, biological, social, and cultural activity that may play a confirmatory role in becoming a ‘mother’ [21, 22]. IPV survivors may doubt their capability to parent and breastfeed [23]. Becoming a mother may increase a woman’s vulnerability, because the IPV no longer only affects her [24]. IPV in pregnancy has been shown to negatively affect women’s body image, which could lead to breastfeeding avoidance [25], and women exposed to IPV tend to use female gender performability (defined as doing what is expected of you in a patriarchal, heterosexual context) to try to protect themselves and their children through their breastfeeding choices [26]. Women may feel ashamed if they choose not to breastfeed or are unable to breastfeed [27]. Research about women’s lived experience of IPV manifestations during breastfeeding is sparse. Therefore, the study aim is to explain and understand IPV manifestations during the breastfeeding period, as experienced by women exposed to IPV.

## Methods

The lifeworld hermeneutic approach used in this study is based on Reflective Lifeworld Research (RLR), which focuses on interpretating and understanding meaning and lived experience in everyday phenomena [28, 29]. The phenomenon under study is *IPV manifestations during the breastfeeding period.* RLR and lifeworld hermeneutics are epistemologically rooted in the phenomenological and hermeneutic ideas of Husserl, Heidegger, Gadamer, and Ricouer [28, 29].

Husserl’s lifeworld theory positions a phenomenon as something experienced by someone, explaining the world as something holding unique meaning for human beings. This emphasises deeper meanings of lived experience shaped by subjective experiences preceding any abstraction or theory. While one’s lifeworld may appear to be shared with others, it is never entirely possible to understand another person [30, 31]. The lifeworld hermeneutic aim to deeply comprehend another person’s existential world requires a scientific approach to understanding, which reflects on how natural attitude and intentionality shape interpretations of other people’s experiences [28]. In this context, ‘natural attitude’ refers to a pre-reflected, taken-for-granted attitude towards the world and the phenomena experienced within it. Intentionality is the fundamental mode of being. People are inherently intentional, immediately perceiving phenomena as meaningful [31].

Heidegger emphasized interpretative ability as an ontological, defining feature of human existence [32]. Gadamer built upon Husserl and Heidegger, claiming pre-understanding’s and traditions’ crucial role in shaping our understanding. He urged researchers to actively reflect on the influences of tradition and pre-understanding in research [33]. Ricoeur noted the phenomenological influence of modern hermeneutics, emphasizing interpretation of lived experience. For Ricoeur, interpretation involves both explanation and understanding [34]. He emphasised intentional explanations, focusing on how and why we experience, instead of on cause and effect [35]. The methodological principles of lifeworld hermeneutics are openness, curiosity, pliability, pre-understanding problematisation, and iterative movement between the whole and parts [28, 29].

### Participants and data collection

Participants were recruited through advertisements in relevant Swedish social media groups, upon approval by their administrators. Forty-nine women participated through written lifeworld stories via the digital tool Sunet Survey, and nine women through lifeworld interviews [29]. The number of women who chose to participate in both venues is impossible to determine, due to anonymisation. Participants’ identities have been protected by limiting personal data collection and offering a choice between written and digital participation. Background data were provided by participants in connection with participation and were only stored and handled at the group level. Participants were 25–45 years old, with education ranging from 3 years of upper secondary school to > 3 years of post-secondary education, and all except 2 women were born in Sweden. All participants reported having lived experience of IPV during breastfeeding by a current or former male partner but did not explicitly define their own sex. Due to correlation between breastfeeding and female biology and gender roles, they are referred to as ‘women’ in the study. There was variation considering IPV types, time elapsed since the experience (0 to > 10 years) and length of partner relationship (< 1 year to > 10 years). The number of children they breastfed or wished to breastfeed in the relationship (1–4 children), and experienced degrees of being hindered from breastfeeding also varied. Interview participants were all born in Sweden, were more likely to have left the relationship, and were generally older and more highly educated than other participants.

Data were collected between June 2022 and August 2023 through written lifeworld stories and lifeworld interviews, beginning with an open-ended question focused on lived experience, in accordance with the aforementioned methodological principles [28, 29]: *Would you please tell me about your experience of exposedness in your partner relationship in connection with breastfeeding or breastfeeding desire?* When deemed appropriate, follow up questions such as: *Can you tell me more about…? When you mention…what does that mean to you?* were used during interviews to deepen and expand meaning within descriptions. Interviews were conducted by telephone or encrypted video meetings according to participant request. They lasted one to two hours and were audio-recorded and transcribed verbatim. Those who participated through written lifeworld stories received contact information for support organisations. The interviewer (IG) lingered after the interviews to assess participants’ needs for support.

### Data analysis

**Fig. 1:**
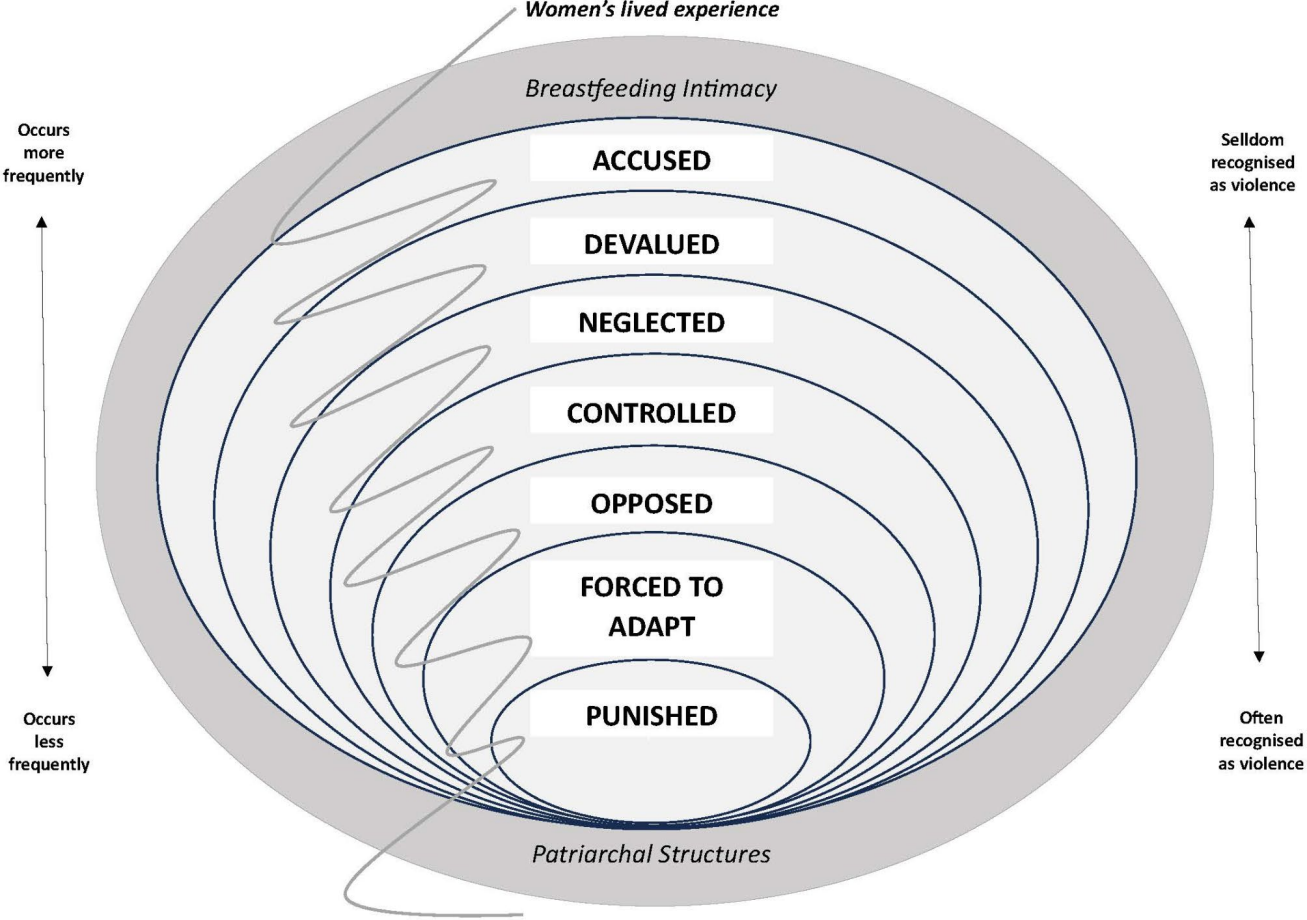
The continuum of IPV manifestations during breastfeeding

In accordance with the lifeworld hermeneutic approach, analysis began by researchers reading and familiarising themselves with the data (the whole). Meanings relevant to the phenomenon were highlighted [28], compared, grouped, and formed into the most meaningful interpreted themes of IPV manifestations (the parts) [29]. The interpreted themes turned out to be intricately intertwined. To be able to develop a main interpretation that meaningfully and abstractedly explained both the intertwining and context of the interpreted themes (the new whole), Kelly’s feminist theory *‘The continuum of violence’* [36] inspired further interpretative analysis. It highlights the intertwining of different types of gender-based violence and concludes that objective grading of seriousness, often found in violence research and legislation, is irrelevant in relation to women’s subjective, context-dependent experience. However, Kelly notes that occurrence seems to correlate with societal acceptance [36]. The theory inspired an understanding of IPV manifestations during breastfeeding as intertwined parts of an IPV continuum, as illustrated in the main interpretation (Fig. 1).

In lifeworld hermeneutics, validation runs parallel to analysis [28]. In this study, adhering to the principles of openness and pliability meant remaining humble and curious regarding the unexpected. Pre-understanding was problematised by making tentative interpretations and reflecting on them and upon one’s own expectations among the other authors. Comparison between data, interpreted themes, and main interpretation counteracted contradictions. The results are illustrated by quotes, sometimes reworded to anonymise participants.

## Results

Women’s lived experience of the study phenomenon is illustrated in seven interpreted themes of IPV manifestations during the breastfeeding period: being *accused*,* devalued*,* neglected*,* controlled*,* opposed*,* forced to adapt*, and/or *punished*. The main interpretation suggests one way to explain and understand how the manifestations are intertwined in a continuum of IPV manifestations during breastfeeding (Fig. 1).

### Being accused

One manifestation of women’s lived experience of IPV during breastfeeding could be interpreted as being accused of counteracting the partner by breastfeeding. Despite partially infant formula-feeding, partners may accuse breastfeeding of sleepless nights, and problems with child weight gain. Breastfeeding women may be accused of complicating the partner-child relationship. They experience these accusations as a socially acceptable excuse to mask their partners’ disinterest in caring for their children. Sometimes women feel forced to compete regarding who is the most important parent. In retrospect, women view accusations surrounding breastfeeding from a broader perspective:

There was jealousy in our relationship and still is today, as the child continues to choose me in front of her father // In the past it was the breastfeeding’s ‘fault’ and now there is nothing to blame, really.

Women understand accusations surrounding breastfeeding to be jealousy of the breastfeeding intimacy within the mother–child dyad. Children’s access to women’s bodies is accused of counteracting partner relationships. Women feel accused of breastfeeding for selfish reasons, spoiling or hurting the children, and counteracting gender equality and independent child development. Being accused of having independently chosen to breastfeed could result in being denied sympathy and support from partners during breastfeeding and breastfeeding difficulties. Sometimes accusations even force the women to reduce or terminate breastfeeding:

I felt pressured not to give the breast. And then he could ask like “Did you give the breast or what??” when I managed to get her [the child] to fall asleep // The breast was some kind of weapon.

### Being devalued

Another manifestation of women’s lived experience of IPV during breastfeeding could be interpreted as being devalued as a breastfeeding woman by the partner. Devaluation seems to be manifested mainly by making women feel like failed breast-feeders by criticizing and mocking their breastfeeding techniques and choices. Women experience that the children’s needs and their own feelings and efforts are devalued:

When I was supposed to have a couple of hours to myself once and because of that, with great effort, had pumped a bottle during the day, he spilled the bottle and could not understand why I was upset.

Women also experience being devalued when partners show embarrassment towards, for instance, long-term or public breastfeeding and low breastmilk production. Partners could value the opinions of relatives and strangers about breastfeeding over the women’s opinions. Devaluation is also experienced as manifesting when partners insinuate that non-functioning breastfeeding or pumping is a feminine failure. A woman with mastitis and an accompanying high fever recounts:

He [the partner] had just gone to work // because it was my job as a woman to take care of this.

Devaluation could manifest in the form of degrading and sexualising comments about the women’s mental or bodily capability to breastfeed. Devaluation is reinforced when partners ignore women’s experiences and pretend their comments are jokes. This may leave the women with feelings of valuelessness:

I also just became a, as I put it, a dairy cow.

### Being neglected

Another manifestation of women’s lived experience of IPV during breastfeeding could be interpreted as being neglected as a breastfeeding woman by the partner. The neglect is manifested through being abandoned with breastfeeding-related tasks, purchases, needs, and feelings. The partners could emphasise their own boundaries and needs yet show irritation when women set their boundaries or express a need for breastfeeding assistance. Partners demanding breastfeeding termination and bottle-feeding could refuse to provide termination support or offer support in an insensitive and selfish way, reinforcing the experience of neglect. Ultimately, women may experience the sole responsibility for breastfeeding, family, and household:

I sat…breastfeeding her…and at the same time // “I’ll have to keep the house warm [lighting the stove], and then I’ll work on his [the partner’s school] assignment, so that he can someday contribute to the economy.”

IPV during breastfeeding could also be manifested through neglect towards children’s breastfeeding needs. Partners could, for instance, refuse to assist hungry children to come to the breast. When partners are dysfunctional as parents, the women’s need for relief is neglected:

[He] was kind of completely inattentive to her needs. Came home with a bile-screaming baby who needed to be fed half an hour ago. You can’t leave your child with such a person.

### Being controlled

Another manifestation of women’s lived experience of IPV during breastfeeding could be interpreted as being controlled by the partner through breastfeeding. Partners seem to consider themselves as having the right to control women’s lives because they are the mothers of their children. They could, for instance, micromanage breastfeeding decisions, demand breastfeeding termination or continuation, and act like they want to take over child feeding. Sometimes only the suspicion that partners dislike breastfeeding makes women deprioritise or hide it. The partners could leave all breastfeeding- and child-related purchases to women living on reduced parental income, resulting in an impaired financial ability to leave the relationship. Isolating control is experienced to be facilitated by the breastfeeding intimacy within the mother–child dyad. Partners could pressure women to move away from loved ones or prevent family visits against the women’s will, posing arguments about small children’s susceptibility to infection. Partners could also control women through manipulation. They may convince both the women themselves, their loved ones, and professionals that they (the partners) are supportive parents while framing the women as mentally unstable. Constant lies, projection of negative characteristics, and questioning of the women’s judgment makes it difficult to identify violence, especially when other people are also deceived. Contradictory behaviour, like praising breastfeeding while simultaneously giving infant formula because breastmilk is insufficient leads to self-doubt and increases women’s vulnerability. A mother of a partly formula-fed child illustrates:

As soon as I left // he rang on the phone // it was a means of power all the time // it didn’t matter if I said it [the child] had just eaten, if he said it was hungry he knew this because he was the baby’s father, and it was my duty as a mother to breastfeed.

Women experience that partners take advantage of their love for their children to further control them. They are sometimes forced to stay in relationships to protect their children from being left alone with their partners, or out of fear their partners will hurt or even kill them if they leave. After having separated, visitations between ex-partners and small children could become dependent on the women’s presence. This gives partners opportunities to continue their control. A woman describes the vulnerability of having to breastfeed in front of the partner who previously abused her sexually:

He was very close to her [the baby] while she was breastfeeding //He noticed that I was very uncomfortable with that closeness. He…like kissed her at the same time. To, well…violate my…integrity.

### Being opposed

Another manifestation of women’s lived experience of IPV during breastfeeding could be interpreted as being opposed by the partner. The partners oppose breastfeeding by counteracting instead of cooperating. Women are questioned in their breastfeeding decisions or pressured to terminate breastfeeding by partners and their relatives. When partners give infant formula against the women’s will, it is experienced as a demonstration of power. Not being allowed to spend time with the children outside of breastfeeding sessions prevents learning about breastfeeding cues. Women experience themselves as being prevented from finding peace during breastfeeding due to partners’ demands to be constantly served. Partners could also provide opposition by prohibiting public breastfeeding or complicating recovery:

She [the baby] preferred sleeping next to me, but her father was furious about it. I was very tired and this time [after breastfeeding] I also fell asleep, then he [the partner] woke me (us) up by yelling at me that I’m sick in the head doing like that [co-sleeping].

Partners’ oppositional behaviour regarding breastfeeding, and demands to terminate breastfeeding, tend to exacerbate after separation. It gives partners new opportunities to oppose women through reports of concern or lengthy legal processes, regardless of earlier disinterest in the children. One woman describes being continuously sued by her ex-partner over the course of several years:

I was just expected to [be able to get to court] all the time // It doesn’t matter if they give 12 time-suggestions. I agree to all but one—then that’s the one he wants. It didn’t matter if it was her [the child’s] time to eat // It’s only this…damned father and his rights // I’ve breastfed [x number] of children. And he [the partner] has been allowed // to be very present in my life even during those periods.

### Being forced to adapt

Another manifestation of women’s lived experience of IPV during breastfeeding could be interpreted as being forced to adapt to the partner’s needs. Partners act as if their needs, views, and well-being are always primary, taking precedence over women’s and children’s needs and well-being. Women experience this narcissistic behaviour as inappropriate for a father but feel forced to adapt due to fear and powerlessness, or because they believe it will result in a more cooperative partner. Over time, this adaptation could become habitual:

I felt sorry for him [the partner] all the time. So, I was like // “I kind of have to think about relieving him” // So I went with…this little skinny baby, back and forth [to another home] // to give him a few days… // relief from…being with me and his daughter—he didn’t actually do anything.

Women who have previously been forced to adapt to their partners in other phases of life experience that violence manifests in new ways during the breastfeeding period. Being responsible for a child makes it harder to focus on one’s partner and household, and women are torn between the needs of their children and their partners. The adaptations seem to correspond to what benefits the partners. When breastfeeding relieves partners, or becomes a tool to punish or limit women, it is accepted or even encouraged. Thus, women are forced into involuntary long-term breastfeeding because partners are unwilling to support termination of breastfeeding if it means they (the partners) will be expected to share in the labour of night-time feeds. Conversely, partners may also demand breastfeeding termination when they feel burdened by its associated demands:

I’ve been thrown between being prompted to breastfeed because it’s cheapest, to being a fat and lazy sow who definitely doesn’t have the right to //relief at night //because I “chose to breastfeed”. // Either I SHOULD breastfeed (like when I’m tired, sick, have to go away) or breastfeeding is a problem because I don’t make money // and tie the baby too strongly to me.

IPV during breastfeeding also manifests through being forced to adapt to a partner’s sexual needs. Some women experience that decreased intercourse frequency postpartum provoke partners and lead to coercion to have unwanted sex. When women are absorbed in initial breastfeeding, and vaginal intercourse is discouraged due to risk of infection, partners may demand anal sex. They could also crave sex during breastfeeding or force women to end breastfeeding sessions to prioritise the partners’ sexual desires. Resistance could result in rape and endanger women and children. Forced adaptation includes feeling constantly sexualised and being unwelcomely touched during breastfeeding:

I feel that my breasts are mainly mine, but they are also my daughter’s dining area//Therefore, I couldn’t see them as a sexual part of my body during the breastfeeding period. It wasn’t respected [by the partner] and I felt uncomfortable, but [I] also froze at times when he made advances. It became uncomfortable when he would start licking my nipples.

### Being punished

The final manifestation of women’s lived experience of IPV during breastfeeding could be interpreted as being punished by the partner. Punishment seems to manifest either through separation from the children or through physical violence. Punishment through separation could involve prohibiting co-sleeping, continuously inviting babysitters over, or giving infant formula. If the women want a divorce, partners could request sole custody or threaten to kidnap the children. The threat of separation becomes a reality when partners leave with breastfeeding children, for hours or even weeks:

Then the child was used as a tool for power // “Now you’re going to have time for yourself. Now I’m going to take the baby and drive away, but I’m not going to say where I’m going or for how long.” // And, I’m like panicking.

Women experience increased punishment through physical violence during breastfeeding. Partners tend to show dislike towards the breastfeeding intimacy within the mother–child dyad and become provoked and violent when breastfeeding prevents women from obeying them. Partners could rapidly switch between kindness and cruelty, claiming to care about the children while, without hesitation, harming their mothers:

Every time that violence occurred was because I resisted him // I thought like a rational person, that he won’t do anything when I’m sitting with the baby [in my arms]…But then he does….

### Main interpretation

To further deepen the analysis of women’s lived experience of IPV manifestations during breastfeeding, the main interpretation aims to explain and understand how these manifestations are intertwined. Kelly’s theory ‘*The continuum of violence*’ [36] has contributed to understanding by visualising that although the IPV manifestations are presented as separable themes, they are intertwined parts of a continuum (Fig. 1). Some IPV manifestations seem more intertwined than others. Being *opposed* could be viewed as an extension of *being neglected*, and both *control* and *devaluation* resemble *being forced to adapt*. Multiple IPV manifestations also occur during single occasions. Being separated from the child by the partner and prevented from breastfeeding could, for instance, simultaneously be experienced as being *punished*,* controlled*,* neglected*, and *opposed*. The continuum is subjective and lifeworld dependent. Objectively similar IPV manifestations may hold different meanings for different women, depending on context and time, for instance. The continuum of IPV manifestations during breastfeeding illustrated in Fig. 1 is consequently nonlinear and multidimensional. The circles representing the IPV manifestations are simultaneously separate units and part of every other circle. IPV manifestations with the highest occurrence in the data are represented by the largest circles. According to Kelly, higher occurrence indicates lower likelihood of being societally recognised as violence [36].

IPV manifestations during breastfeeding do not exist in a vacuum. To grasp the continuum, understanding of its context is central. Supported by Kelly [36], the continuum of IPV manifestations during breastfeeding is interpreted as existing because patriarchal societal structures preserve and uphold female oppression and objectification. In this context, men have the power to set the rules, and demand that women exist to satisfy them. The intimate nature of breastfeeding seems to provoke this idea by changing the intersubjective power balance of the partner relationship, thereby questioning the partner’s supremacy. This results in competitive behaviour, accusations, neglect, and devaluation of women, children, and breastfeeding. Women could feel forced to adapt to their partners to protect themselves and their children from exposure to violence, even after a separation. Having a mutual child seems to mean living in a never-ending partner relationship. The boundness to the children that breastfeeding implies makes it harder to adapt to one’s partner’s demands and risks increasing IPV. Breastfeeding intimacy could also make women vulnerable to attempts to isolate them from their children. Patriarchal structures and breastfeeding intimacy are understood as the context in which the IPV continuum exists during breastfeeding and are illustrated in Fig. 1 by a surrounding circle.

## Discussion

### Results discussion

This study shows that women’s lived experience of IPV during breastfeeding manifests as a multidimensional, lifeworld-dependent continuum of being accused, devalued, neglected, controlled, opposed, forced to adapt, and/or punished. The study is unique, as women’s experience of the phenomenon do not appear to have been studied previously in the breastfeeding context. However, a recent Nordic qualitative review including women’s experience of the impact of violence on pregnancy and childbirth, supports the study’s results by showing that women experience themselves as being controlled, isolated, rejected, manipulated, powerless, oppressed, afraid, sexually coerced, and forced to adapt to survive and protect the child from harm [37]. Additionally, mother’s experience of parenting in the IPV context shows similarities with the result by showing that partners’ opposing and controlling behaviour forces women to prioritise their partner in front of their child. Women also feel disrespected and taken-for-granted in the mothering role [38]. The current study contributes to an understanding of the nature of IPV in the unique context of breastfeeding.

According to Kelly, all women risk experiencing IPV. No clear demarcation exists between victims and “women in general” [36]. Societal gender constructions and patriarchal power structures normalise gender-based violence in every woman’s life [39] and limit her freedom [36]. However, age, divorce, financial difficulties [40], disability [41], and immigration have been highlighted as examples of being especially vulnerable to IPV, by implying higher risk or more severe consequences [42]. This study contributes by emphasising breastfeeding women as especially vulnerable to IPV. The transition to motherhood [43] and initial breastfeeding are existentially life changing, and breastfeeding difficulties could be experienced as motherhood failure [21, 44]. During initial breastfeeding, women’s bodies adapt physiologically to the children [45]. Additionally, the current study shows that the unique breastfeeding intimacy within the mother–child dyad, and the physical and mental closeness it implies, may change the power structures of the partner relationship, potentially making women more vulnerable to manipulation by partners. In this way, becoming a mother and initiating breastfeeding while experiencing IPV could be interpreted as a triple vulnerability, inseparably intertwined with female embodiment. According to Young, female breasts are, in our western culture, construed as sexual objects belonging to the partner. Breastfeeding challenge this construct, as the breasts may instead be perceived as being ‘owned’ by the child [46]. This could explain at least in part the jealousy women in our study perceived in their partners, and why IPV occurs. Independent of IPV experience, childbearing imposes a risk of decreased partner relationship quality [47]. Formerly gender-equal partners risk becoming unequal parents because gendered, embodied ideals of parenting, pregnancy, and breastfeeding mostly affect women [48]. Partner support is important for breastfeeding [49, 50]. However, if partners feel excluded and incapable [51] they may blame breastfeeding [48]. These difficulties in ‘normal parenthood’ during the breastfeeding period easily raise questions about the distinction between ‘normal vulnerability’ and vulnerability due to exposure to IPV. This study contributes by questioning the existence and meaning of such a distinction. The continuum shows the diversity of women’s lived experience of IPV during breastfeeding in a patriarchal society. In accordance with a caring science perspective, it calls for a shift of focus from objective definitions to the subjective experience of women exposed to IPV [29].

Care means decreasing suffering and increasing health and well-being [52]. Health and wellbeing are subjective and changeable experiences of holistic balance in all parts of life, linked to an ability to reach one’s life goals and experience coherence and meaning [53]. In accordance with this study, IPV has been shown to oppose women’s empowerment and sense of purpose [23] and continues past relationship termination [41]. Nursing research and literature on IPV and mothering shows a cisgender, heterosexual, and individual focus, while IPV is structural and complex with long-term, post-separation effects [54]. The results of this study show that women experiencing IPV during breastfeeding suffer physically, mentally, and existentially. According to Galvin and Todres, the intersubjective suffering dimensions of aversion, isolation, and persecution could mean being victimised by another person and finding no escape. They stress the importance of carers recognising different dimensions of suffering to enable empathic care where vulnerability is recognised and shared [55]. The results show that IPV during breastfeeding may manifest differently than it does during pregnancy, stressing the importance of holding dialogues about IPV postpartum, to decrease the risk of it going unnoticed by carers. The study results prompt carers to pay attention to IPV manifestations during breastfeeding and consider questions such as: is the breastfeeding woman focusing on the needs of the child or of her partner? Is the partner constantly involved in detailed breastfeeding decisions (control) or completely absent (neglect)? Do breastfeeding problems arise because the woman is prevented from learning breastfeeding cues? Carers risk exacerbating the situation by, for instance, agreeing when partners accuse breastfeeding of complicating the partner-child relationship, or by advising the new family to reduce social contact. In a Nordic, ‘gender-equal’ context, where claims of equality in relation to child feeding is common, awareness that it may conceal a partner’s intention to reduce the breastfeeding intimacy within the mother–child dyad, as well as the child’s access to the woman’s body, is crucial.

### Methodological considerations

Lifeworld hermeneutics are guided by the quality criteria of objectivity, generalisation, and validity [29]. Objectivity—considering openness to the ‘otherness’, as described by Gadamer [33] —was challenging due to IPV stigma. Not all women experiencing IPV define themselves as having been exposed [56] because patriarchal power structures and men’s interpretative supremacy in society, laws, and regulations complicate women’s understanding of their own situations [36]. In participant recruitment and data collection, we therefore asked for experience of exposedness in partner relationships instead of experience of IPV. During interviews, the women’s experience of exposedness in relation to IPV were deepened. In accordance with the methodological approach, this contributed to rich variation in reported phenomena [29]. Quotes from various written and verbal lifeworld stories are included in the results. The interviews were richer in words and meaning but fewer in number. The written lifeworld stories framed the interviews and contributed to variation in phenomena.

A study limitation is the homogeneous group of participants in terms of, for example, origin and sexual orientation. This occurred despite adopting diversity measures like using the gender-neutral word ‘partner’, searching for participants through various social media groups, and offering both written and oral participation. Generalisation in lifeworld hermeneutics is possible through abstract movement between parts and whole and the construction of a main interpretation is a study strength. However, the results need to be considered in context [29], and further research is required to elucidate phenomena occurring in, for instance, the context of same-sex relationships.

Openness is a core validity prerequisite in lifeworld hermeneutics [28, 29] and guided the analysis of the interpreted themes of IPV manifestations. During further analysis and development of the main interpretation, a need for theoretical support to fully understand the intertwining of the interpretations became evident. Sticking to a certain theory could be considered a limitation in terms of study openness. However, in a lifeworld hermeneutical sense, validity is not achieved through excessive use of rules and methods, but by openness towards the phenomenon [28, 29]. Using theory as interpretative support, especially in main interpretations, could be helpful and even desirable in reducing the influence of pre-understanding [28]. The continuum of violence theory [36] made interpretations more meaningful by offering structure in the main results and an explanation for interpretation’s intertwining and subjectiveness. Feminist theory contributed by highlighting women’s lived experience, in accordance with lifeworld hermeneutics.

## Conclusions

This study shows that women experiencing IPV during breastfeeding feel accused, devalued, neglected, controlled, opposed, forced to adapt, and/or punished. The IPV manifestations form a multidimensional, lifeworld-dependent continuum existing in a patriarchal context. The breastfeeding intimacy within the mother–child dyad and changed intersubjective power balance in the partner relationship seem to provoke partners. The study fills a knowledge gap and contributes by clarifying that the breastfeeding period implies vulnerability in relation to IPV and its consequences. The vulnerability seems inseparably intertwined with motherhood and female embodiment. By highlighting women’s lived experience, care and support may improve, supporting the health and well-being of women exposed to IPV.

## Data Availability

Data are not openly available due to sensitivity reasons. They are available from the corresponding author upon reasonable request. Data are stored in controlled access data storage at the University of Borås, Sweden.

## Abbreviations

IPV: Intimate Partner Violence
RLR: Reflective Lifeworld Research
WHO: World Health Organisation
